# Evidence of Introgression of the *ace-1^R^* Mutation and of the *ace-1* Duplication in West African *Anopheles gambiae* s. s

**DOI:** 10.1371/journal.pone.0002172

**Published:** 2008-05-14

**Authors:** Luc Djogbénou, Fabrice Chandre, Arnaud Berthomieu, Roch Dabiré, Alphonsine Koffi, Haoues Alout, Mylène Weill

**Affiliations:** 1 Institut de Recherche pour le Développement, Cotonou, Benin; 2 Centre de Recherche Entomologique de Cotonou (CREC), Cotonou, Benin; 3 Université Montpellier 2, Montpellier, France; 4 CNRS, Institut des Sciences de l'Evolution, Equipe Génétique de l'Adaptation, Montpellier, France; 5 Institut Régional des Sciences de Santé, Laboratoire d'Entomologie et de Parasitologie, Bobo-Dioulasso, Burkina Faso; 6 Institut Pierre Richet (IPR)/Institut National de Santé Publique, Abidjan, Côte d̀Ivoire; University of Sydney, Australia

## Abstract

**Background:**

The role of inter-specific hybridisation is of particular importance in mosquito disease vectors for predicting the evolution of insecticide resistance. Two molecular forms of *Anopheles gambiae* s.s., currently recognized as S and M taxa, are considered to be incipient sibling species. Hybrid scarcity in the field was suggested that differentiation of M and S taxa is maintained by limited or absent gene flow. However, recent studies have revealed shared polymorphisms within the M and S forms, and a better understanding of the occurrence of gene flow is needed. One such shared polymorphism is the G119S mutation in the *ace-1* gene (which is responsible for insecticide resistance); this mutation has been described in both the M and S forms of *A. gambiae* s.s.

**Methods and Results:**

To establish whether the G119S mutation has arisen independently in each form or by genetic introgression, we analysed coding and non-coding sequences of *ace-1* alleles in M and S mosquitoes from representative field populations. Our data revealed many polymorphic sites shared by S and M forms, but no diversity was associated with the G119S mutation. These results indicate that the G119S mutation was a unique event and that genetic introgression explains the observed distribution of the G119S mutation within the two forms. However, it was impossible to determine from our data whether the mutation occurred first in the S form or in the M form. Unexpectedly, sequence analysis of some resistant individuals revealed a duplication of the *ace-1* gene that was observed in both *A. gambiae* s.s. M and S forms. Again, the distribution of this duplication in the two forms most likely occurred through introgression.

**Conclusions:**

These results highlight the need for more research to understand the forces driving the evolution of insecticide resistance in malaria vectors and to regularly monitor resistance in mosquito populations of Africa.

## Introduction

Understanding the dynamics of genes associated with insecticide resistance between closely related taxa is a key component in establishing effective long-term resistance management strategies. Adaptive genes such as those producing insecticide resistance allow for the ability to detect rare hybridization events within a group of related taxa. There are three major target sites for most insecticides: the g-aminobutyric acid (GABA) receptor is the target of cyclodiene insecticides, the voltage-dependent sodium channel is the target site for DDT and pyrethroids, and acetylcholinesterase (AChE1, EC 3.1.1.7) is quasi-irreversibly inhibited by organophosphorous and carbamate compounds, which are substrate analogues. In Africa, propoxur resistance was first detected in a population of *Anopheles gambiae* Giles from Côte d'Ivoire [1]. Insensitive AChE1 was next confirmed as the resistance mechanism [2]. As in *Culex pipiens* L., *Anopheles albimanus* Weidemann and *Culex vishnui* Theobald [3]–[6], the resistance in *A. gambiae* results from a single mutation in the *ace-1* gene that leads to a Gly-to-Ser substitution at position 119 (according to the *Torpedo californica* nomenclature) [7]. The resistant allele *ace-1^R^* confers resistance to organophosphates and carbamate compounds. This resistance potentially represents a threat to the implementation of malaria prevention programmes based on the use of insecticides.

The *Anopheles gambiae* complex includes some of the most important malaria vector species of Sub-Saharan Africa. Genetic differentiation occurs within this highly polymorphic complex that is subdivided into five cytoforms. These cytoforms differ in their arrangements of chromosomal inversion and appear more or less genetically isolated in the field [8], [9]. In addition, studies using molecular markers such as X-linked ribosomal DNA revealed the presence of two distinct molecular forms within *A. gambiae* s.s. These forms have been referred to as M and S forms [10] and are now considered as units of an on-going incipient speciation process [11]. A high deficit of M/S hybrids observed in the field has been attributed to assortative mating because hybrids are readily obtained in the laboratory [11]–[17]. Thus, it has been suggested that the differentiation into M and S taxa is maintained by limited or absent gene flow [11]. However, the absence of gene flow is still debated because recent work using molecular markers showed that differentiation between the M and S forms is only present in few regions of the genome: a small region close to the centromere of the 2^nd^ chromosome, and the centromeric region of the X chromosome that contains the rDNA that defines these molecular forms [14], [18]–[20]. Evolutionary histories of closely related species are often complicated by the existence of shared polymorphisms (inherited from their common ancestor) as well as possible ongoing gene flow between them [21]. Today, the debate over whether the limited differentiation among molecular forms of *A. gambiae* s.s. reflects insufficient time for more differentiation to have occurred versus introgression is not resolved. However, some recent introgressive hybridization phenomena within *A. gambiae* s.s. are well documented [14]–[16].

Recently, studies have been conducted to estimate the distribution of the *ace-1^R^* G119S mutation in populations of *A. gambiae* s.s. from Burkina Faso [22]. The mutation was found in both M and S forms where they were sympatric. It is important to determine how often the G119S resistance mutation occurs in natural populations in order to understand its evolutionary history within the *A. gambiae* s.s. complex. In the *Culex pipiens* mosquito complex, the G119S mutation appeared several times independently. Two distinct mutation events were first reported in *C. pipiens* and *C. quinquefasciatus* species [5]. A third event was next described in *C. quinquefasciatus* from China [23] and a polymorphic intron sequence located upstream of exon 3 revealed two more events within *C. quinquefasciatus* [24].

In this paper, we investigated whether the *ace-1^R^* mutation has arisen in M and S molecular forms through two independent mutations or through genetic introgression. We analysed the polymorphism of the DNA sequence associated with the G119S mutation (both introns upstream and downstream from exon 3) in several resistant and susceptible individuals of *A. gambiae* s.s of both M and S molecular forms from Burkina Faso, Benin and Côte d'Ivoire. Our data clearly indicate that the G119S mutation was a unique event and that genetic introgression explains the observed distribution of the G119S mutation within the two forms.

## Materials and Methods

### Mosquito samples

**Figure 1:**
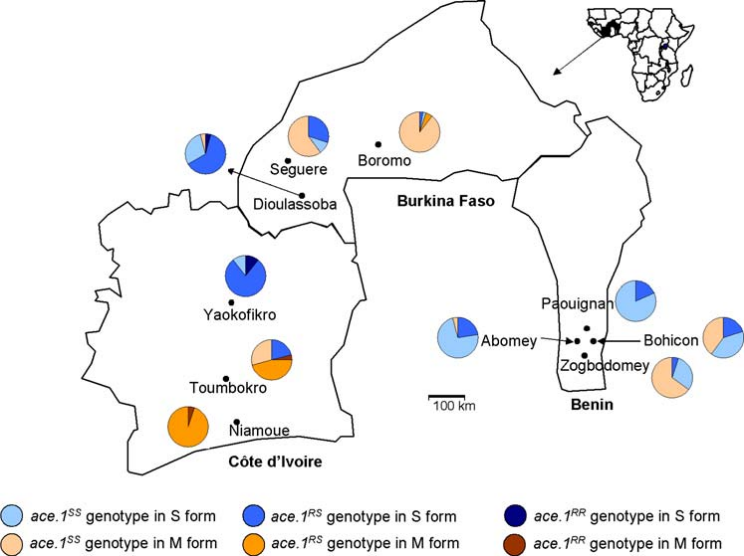
Map of Benin, Burkina Faso and Côte d'Ivoire showing the study sites. (•): study locality. Allelic frequencies of the *ace-1* ^R^ mutation in *A. gambiae* s.s. are indicated in each molecular form M and S.

Larvae were collected at several locations from Burkina Faso, Benin and Côte d'Ivoire (Figure 1) and reared in the laboratory until emergence. Adult *A. gambiae* s.l. were sorted from other anophelines according to morphological identification keys [25] and were kept at −20°C for molecular analysis.

### DNA extraction, PCR identification of *A. gambiae* M and S forms, and *ace-1* genotyping

Total genomic DNA was extracted from individual mosquitoes following a slightly modified procedure described by Collins [26]. Species in the *A. gambiae* complex and molecular forms of *A. gambiae* s.s. were identified using the methods described by Scott [27] and Favia [10], respectively.

For *ace-1* genotyping, 1–10 ng of genomic DNA was amplified with the primers Ex3AGdir and Ex3AGrev [6]. PCR was conducted in 25 µL volumes containing 2.5 µL 10× *Taq* DNA polymerase buffer, 200 µM deoxynucleoside triphosphate (dNTP), 0.1 U *Taq* DNA polymerase (Qiagen, France) and 10 pmol of each primer. PCR conditions included an initial denaturation step at 94°C for 5 min followed by thirty five cycles of 94°C for 30 s, 54°C for 30 s, and 72°C for 30 s, and a final extension at 72°C for 5 min. Fifteen µL of the PCR product were digested with 5 U of *Alu*I restriction enzyme (Promega, France) in a final volume of 25 µL. The restriction products were fractionated on 2% agarose gels (Agarose, MP, Sigma) with TBE buffer (0.089 M Tris, 0.089 M boric acid and 0.5 M EDTA, pH 8.0), stained with ethidium bromide, and observed under UV light.

### Partial sequences of *ace-1* alleles

DNA from single mosquitoes was amplified using two specific primers: AgEx2dir1 5′-AGG TCA CGG TGA GTC CGT ACG A-3′ and AgEx4rev2 5′-AGG GCG GAC AGC AGA TGC AGC GA-3′ generating an 817 bp fragment that included part of exon 2, intron 2, whole exon 3, intron 3 and part of the exon 4. PCR was carried out with ∼20 ng of genomic DNA, 10 pmol of each primer, 10 µm of each dNTP, 2.5 units of Taq polymerase (High Fidelity) in 1× reaction buffer (Tris–HCl [pH 9.0; 75 mM], (NH_4_)2SO_4_ [20 mM], Tween 20 [0.1 g.liter^−1^], and MgCl_2_ [1.25 mM]) for a final volume of 50 µL. The PCR mixture was subjected to 30 amplification cycles (93°C for 30 s, 56° for 30 s and 72°C for 50 s). PCR products were purified using the QIAquick Gel Extraction Kit (QIAGEN). For homozygous resistant mosquitoes, the purified PCR product was directly sequenced. For heterozygous and susceptible homozygous mosquitoes, the purified PCR product was cloned using the TOPO_ Cloning Kit (Invitrogen, Paisley, UK) according to the manufacturer instructions. Clones were then screened for the presence of the G119S substitution [6]. At least six clones were sequenced for each mosquito, i.e., three susceptible allele sequences and three resistant allele sequences for heterozygotes and six susceptible allele sequences for susceptible homozygotes. Sequencing was conducted on an ABI Prism 310 sequencer (BigDye Terminator Kit, Applied Biosystems, Foster City, CA). Both strands of the genome fragment were sequenced for each clone using the primers AgEx2dir1 and AgEx4rev2.

### Sequence analysis

Sequences were aligned with Multalin software [http://www.prodes.toulouse.inra.fr/multalin/multalin.html] [28] using the Kisumu strain as a reference. Similarity between various sequences was assessed with ClustalW [29]. Sequence polymorphism and nucleotide diversity were analysed with DnaSP, v.4.10.3 [30] using Nei's p index [31], [32]. Deduced amino acid sequences were obtained with ClustalW [29] to determine whether the mutations identified were synonymous. When a mutation was not synonymous, the position of the new amino acid was sought on a 3-dimensional model of the *Torpedo californica* AChE (pdb 1EA5), using Swiss-PdbViewer v. 3.7 [33] to determine whether this mutation might be located at a site crucial for protein activity.

## Results

### Distribution of the *ace-1^R^* mutation in the populations sampled

Two hundred and eighty two mosquitoes from Burkina Faso (n = 83), Benin (n = 135) and Côte d'Ivoire (n = 64) were assayed for molecular form and analyzed for their *ace-1* genotype. The M and S molecular forms are sympatric in seven of the ten localities selected for this study (Table 1). The G119S mutation was distributed at various frequencies in the S and M forms, depending on the location. This mutation was not detected in the M form of *A. gambiae* s.s from Benin (0% compared to 11% in the S form) and was weakly detected in Burkina Faso (2% in M form compared to 31% in the S form). However, it was strongly detected in Côte d'Ivoire (44% of the M form and 50% of the S form). The number of homozygotes for the *ace-1^R^* mutation was very low, even in localities from Côte d'Ivoire, despite the high frequency of resistant heterozygotes in some samples (Figure 1).

**Table 1:** Genotype distribution of M and S molecular forms in different locations.

Locality	*ace.1* genotype	sample	
		S Sample	M Sample
Benin			
• Abomey	RR		
	RS	16 (2)	
	SS	52 (1)	3
• Bohicon	RR		
	RS	1(1)	
	SS	2 (1)	2 (2)
• Paouignan	RR		
	RS	7 (3)	
	SS	32	
• Zogbodomey	RR		
	RS	1 (1)	
	SS	6	13 (3)
Burkina Faso			
• Boromo	RR		
	RS	1(1)	2 (2)
	SS	1(1)	35 (2)
• Dioulassoba	RR	1	
	RS	15 (2)	
	SS	7	1
• Seguere	RR		
	RS	6 (2)	
	SS	2 (1)	12 (2)
Côte d'Ivoire			
• Yaokofikro	RR	2	
	RS	15 (1)	
	SS	3	
• Toumbokro	RR		1
	RS	5 (1)	11
	SS		7 (1)
• Niamoue	RR		1 (1)
	RS		19 (2)
	SS		

The number of individuals sequenced is shown into brackets.

### Variability of Susceptible and Resistant Alleles

**Figure 2:**
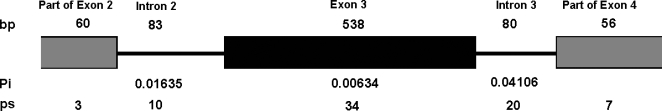
Genomic structure and polymorphism of partial sequences of the *A. gambiae ace-1* gene. bp: number of bases for each sequence. Pi: Nucleotide diversity. ps: Number of polymorphic sites.

**Figure 3:**
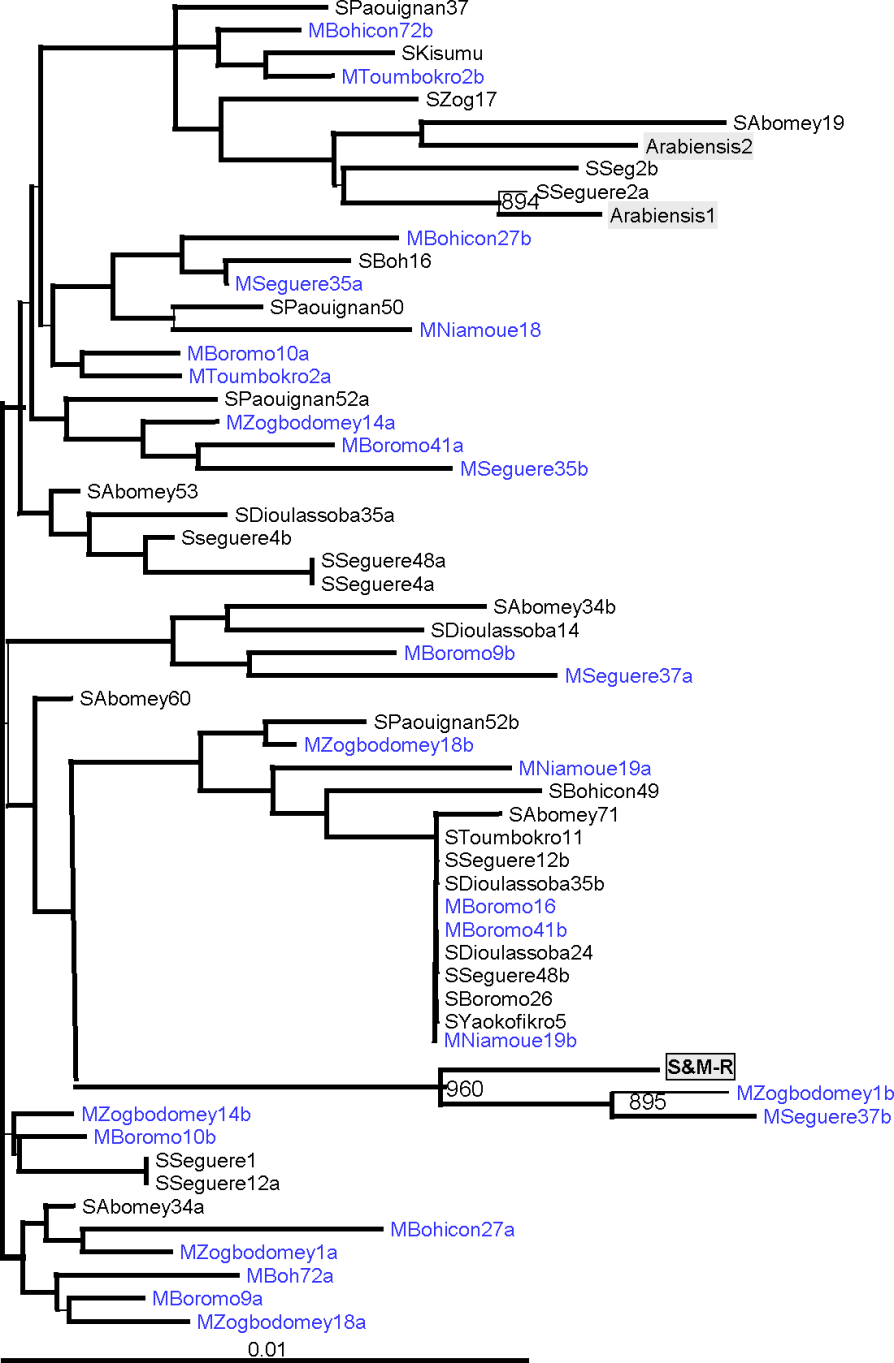
Genetic distance tree of *ace-1* alleles in the M and S forms of *Anopheles gambiae* s.s. Samples were named as followed: S or M before the name of the locality and individual number. Where two distinct susceptible alleles were present in one mosquito, these are noted by “a” and “b” Blue characters are M form samples and black are S form. Variability of the single resistant allele M&S-R and all susceptible alleles of each form are presented and only supported bootstraps are shown. Two sequences of *Anopheles arabiensis* are included and highlighted in grey. For each allele, the sequence of *ace-1* considered encompassed part of exon 2, intron 2, exon 3, intron 3, and part of exon 4. The G119S mutation, selected for resistance to carbamate and organophosphate, has been removed to take only neutral variation into account.

To search for polymorphism in the *ace-1* gene, 33 individuals of *A. gambiae* s.s. from several localities of Benin, Burkina Faso and Côte d'Ivoire, and two *Anopheles arabiensis* Patton from Bohicon (Benin), were analysed. Sequencing of susceptible and resistant *ace-1* alleles was conducted on an 817 bp fragment from M form 15 individuals and 18 S form individuals (Table S1). Forty-eight distinct alleles were identified from the 35 individuals studied (33 *A. gambiae* s.s. and two *A. Arabiensis*; Table S1, with identical alleles removed). The sequence analysis showed that all sequences carrying the resistance mutation (i.e., 14 S and 5 M individuals) were identical regardless of their geographical origin and molecular form; these were annotated as M&S-R (Table S1). We measured the variability of the *ace-1* alleles in our sample. Seventy-four positions exhibited polymorphism: 3, 10, 34, 20 and 7 polymorphic positions in exon 2, intron 2, exon 3, intron 3, and exon 4, respectively (Figure 2). A 3 bp insertion was detected in intron 2 in the sequence of some M and S individuals (Table S1). In addition to the mutation at position 363 corresponding to the G119S substitution, 42 variable positions were shared by at least two individuals. The nucleotide diversity of the coding exon 3 was estimated as Pi: 0.00634; no nucleotide position was found specific to the M or S form. A distance tree constructed (Figure 3) showed low bootstrap values although a few nodes were supported. The distance tree did not reveal any organisation associated with geographical origin or with a particular molecular form. Moreover, the two sequences from *A. arabiensis* segregated among *A. gambiae* s.s. alleles without forming an identifiable outgroup. Among the susceptible alleles, none was identical to that of the resistant allele (excluding the resistant mutation). However, two alleles belonging to the M form, M-Zogbodomey1b and M-Seguere37b, were close to the resistant one (Figure 3). M-Zogbodomey1b differs from the resistant allele by 8 mutations, and M-Seguere37b differs by 11 mutations plus a 3 bp insertion in intron 2. In addition, a susceptible allele was shared by several mosquitoes belonging to both forms (Figure 3).

### Protein Sequence Variability

**Figure 4:**
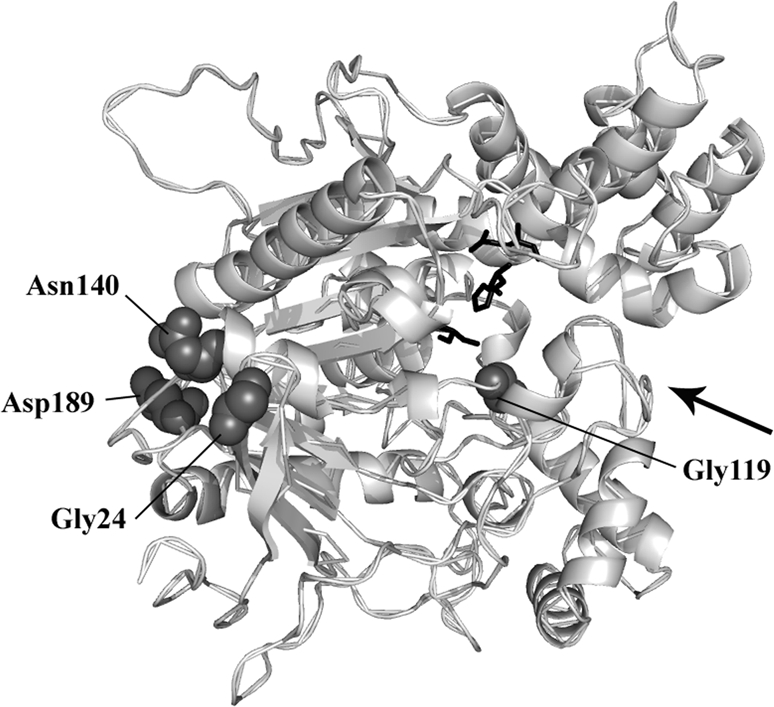
Structural models of *A. gambiae* AChE1 (pdb : 2AZG) used to locate amino acids substitutions found in susceptible *A. gambiae* populations from Bénin. The three substitutions (G24, N140, D189) are represented in dark grey (Van der Waals grey spheres) as well as the 119 position involved in insecticide resistance. The catalytic triad (S200, E327 and H440) appears as black sticks. The arrow shows the entrance of the catalytic gorge. The backbone of the enzyme structure is rendered as a ribbon with secondary structure.

The comparison of the coding region of each susceptible sequence to the single resistant one (651 bp) revealed 45 polymorphic sites (excluding the G119S mutation) but no insertions/deletions. Nucleotide diversity was low (Pi≃0.00810). Only three non-synonymous mutations were identified in three sequences among the 48 distinct alleles analysed (Table S1). Using the *Torpedo* nomenclature [34], we observed the following substitutions: Gly to Ser at position 24 in a Toumbokro2b individual, Asn to Ser at position 140 in a Bohicon72a individual and Asp to Val at position 189 in a Niamoue19 individual. The AChE1 structural model based on that of *Torpedo californica* (PDB: 1EA5) shows that the amino acid substitutions are located at the periphery of the enzyme, far from the active site and its entrance. These amino acids are not likely to have any function in the catalytic activity of AChE1 (Figure 4).

### Heterozygotes harboring three alleles suggest *ace-1* duplication

For each resistant heterozygote, at least 3 susceptible clones were sequenced in effort to avoid any polymerase mistakes. Four mosquitoes from Burkina Faso and one from Côte d'Ivoire contained three different alleles (one resistant and two distinctly different susceptible alleles). The PCR, cloning and sequencing were repeated to avoid any possibility of contamination, and more susceptible clones were sequenced to confirm the first results. For the heterozygote M-Boromo41 and M-Niamoue19, the two susceptible copies differed by 13 positions and 6 positions, respectively. For S-Seguere48, S-Dioulassoba35 and S-Seguere12, the two susceptible copies differed by 14 positions, 13 positions and 12 positions, respectively. In addition, the comparison of susceptible alleles among these mosquitoes showed that they shared one identical susceptible sequence (Figure 3). This common sequence is also shared by other mosquitoes (Figure 3).

## Discussion

In this study, we attempted to describe how the *ace-1^R^* mutation occurred in the two molecular forms M and S of *A. gambiae* s.s., i.e., whether by independent evolutionary gains or by introgression. The sequence of the resistant allele was identical in all homozygous and heterozygous individuals regardless of their geographic origin or molecular form. Although the recorded nucleotide diversity of *ace-1* gene was low in coding sequences (Pi = 0.00810), it increased significantly when non-coding introns were considered (Pi = 0.01224). The probability of the G119S mutation to have been gained twice independently at the same position on a same susceptible allele in both molecular forms is extremely low. Thus, the presence of the *ace-1^R^* mutation in both M and S forms probably resulted from introgression. However, an alternative explanation is that sibling species sharing the same allele could also reflect ancestral retention. This has been documented in other groups for mtDNA genes [35]–[37], and recently in the *A. gambiae* complex [38]. If this was the case, shared haplotypes will pre-date differentiation of S and M forms and will have been maintained by chance in the two forms through incomplete lineage sorting.

The resistant allele *ace-1^R^* has been thoroughly studied in *Culex pipiens* mosquitoes and was found to be associated with a high fitness cost [39]–[43]. The cost probably results from the excess of ACh in synapses because the enzymatic activity of the AChE1R is more than 60% lower than that of the AChE1S [44]. The activity of AChE1R was also found to decrease in *A. gambiae* resistant mosquitoes and a high fitness cost is probably also associated with AChE1R resistance in this species [45]. Thus, because of high fitness costs, G119S resistant mosquitoes cannot be detected after a few generations in the absence of selection pressure by organophosphorous or carbamate insecticides [24]. Since the use of pesticides is very recent (i.e., last few decades) compared to the differentiation of M and S forms, it is likely that the G119S mutation occurred in one form and introgressed in the other. From the analysis of sequence polymorphisms, it is difficult to determine in which form the mutation first occurred because there were no sequences specific to one form. However, because the resistant sequence does form a significant cluster with two susceptible sequences that are both from the M form, we can speculate that the mutation probably occurred first in the M form.

In our study, we also showed that some mosquitoes carried three different *ace-1* alleles: one resistant and two distinct susceptible alleles (see Figure 3). This observation demonstrates the existence of a duplication of the *ace-1* gene that resulted in one susceptible copy and one resistant copy, both of which are on the same chromosome. Such a duplicated *ace-1^D^* allele has been described several times in *Culex pipiens* and results in a “fixed” heterozygous phenotype that displays the same resistance level but with a reduced fitness cost compared to the homozygous resistant phenotype [24], [46]–[47]. The deficit of RR homozygotes in the analysed samples also suggests the presence of the duplication. The sequence comparison of susceptible alleles showed that the mosquitoes with three different alleles of *ace-1* all shared one susceptible sequence (Figure 3), indicating that the mosquitoes contain the same duplicated allele. Furthermore, this duplication was observed in individuals of both molecular forms (Boromo41 and Niamoue19 individuals belong to the M form while Seguere48 and Dioulassoba35 belong to the S molecular form; Table S1). Thus, this suggests that the *ace-1* duplication also occurred in both forms through introgression. Monitoring the frequency of the *ace-1^R^* allele in both the M and S forms of *A. gambiae* s.s will greatly improve our knowledge of such gene duplications evolution.

Currently, there are several, ongoing projects to develop new strategies of vector control with the aim of improving resistance management. It is clear that resistance towards pyrethroids is widespread in Africa and interest in using IRS (Indoor Residual Spraying) to control malaria vectors is resurging. This IRS strategy is preferentially based on the use of organophosphates and carbamates, either alone or in combination with pyrethroids, and the spread of the *ace-1^R^* mutation could represent a major threat to the effectiveness of this strategy. Furthermore, the presence of the *ace-1* duplication could impede strategies based on alternating compounds in space or time. Indeed, such strategies rely on the fitness cost associated with resistance that could decrease in presence of the duplication [24]. Understanding or predicting the spread of insecticide resistance genes into mosquito populations of the *A. gambiae* complex will be crucial for the development of effective methods to control the main malaria vector in Africa.

## Supporting Information

Table S1 (0.63 MB DOC)
