# Supplementary material for: Evidence of Introgression of the ace-1R Mutation and of the ace-1 Duplication in West African Anopheles gambiae s. s
Source: PLoS One. 2008 May 14;3(5):e2172. doi: 10.1371/journal.pone.0002172 (PMC2377098; doi:10.1371/journal.pone.0002172)
Supplement: Table S1 — (0.63 MB DOC) [file pone.0002172.s001.doc]

|  | | | Exon 2 | | | | |  | | Intron 2 | | | | | | | | | | | | | | | | | | | | | | | | | |  | | | Exon 3 | | | | | | | | | | | | | | | | | | | | | | | | | | | | | | | | | | | | | | | | | | | | | | | |  | |
| --- | --- | --- | --- | --- | --- | --- | --- | --- | --- | --- | --- | --- | --- | --- | --- | --- | --- | --- | --- | --- | --- | --- | --- | --- | --- | --- | --- | --- | --- | --- | --- | --- | --- | --- | --- | --- | --- | --- | --- | --- | --- | --- | --- | --- | --- | --- | --- | --- | --- | --- | --- | --- | --- | --- | --- | --- | --- | --- | --- | --- | --- | --- | --- | --- | --- | --- | --- | --- | --- | --- | --- | --- | --- | --- | --- | --- | --- | --- | --- | --- | --- | --- | --- | --- | --- | --- | --- | --- |
|  | | | 111 | 114 | | 129 | |  | | 26 | | 27 | | 38 | | 44 | | 48 | | 50 | | 51 | | 52 | | 55 | | | 56 | | 59 | | 71 | | 76 | | |  | | 2 | | 14 | | 21 | | 47 | | 56 | | 78 | | 101 | | 116 | | 122 | | 131 | | 134 | 135 | | 140 | | 200 | | 218 | | 236 | | 266 | | | | | 278 | 317 | | 323 | | 335 | | 362 | | **363** | |  | |
| Kisumu | | | T | G | | C | |  | | G | | A | | G | | T | | A | | * | | * | | * | | T | | | G | | C | | G | | C | | |  | | C | | C | | C | | C | | C | | G | | C | | C | | G | | G | | G | T | | G | | C | | C | | C | | G | | | | | G | A | | G | | G | | C | | G | |  | |
| S&M-R | | | - | - | | A | |  | | - | | - | | A | | - | | - | | * | | * | | * | | C | | | - | | - | | - | | - | | |  | | - | | - | | - | | - | | - | | - | | - | | - | | - | | - | | - | C | | - | | - | | - | | - | | - | | | | | - | G | | - | | - | | - | | **A** | |  | |
| Arabiensis1 | | | - | - | | - | |  | | - | | - | | - | | - | | - | | * | | * | | * | | - | | | - | | - | | - | | - | | |  | | - | | T | | - | | - | | - | | - | | - | | - | | - | | - | | - | C | | - | | - | | - | | - | | - | | | | | C | G | | A | | - | | - | | - | |  | |
| Arabiensis2 | | | - | - | | - | |  | | - | | - | | A | | - | | - | | * | | * | | * | | - | | | - | | - | | - | | - | | |  | | T | | - | | - | | - | | - | | - | | - | | - | | - | | - | | - | C | | - | | - | | - | | - | | - | | | | | - | - | | - | | - | | G | | - | |  | |
| MBoromo41 | | | - | - | | A | |  | | - | | - | | A | | - | | - | | * | | * | | * | | C | | | - | | A | | - | | - | | |  | | - | | - | | - | | - | | - | | - | | - | | - | | - | | - | | - | C | | - | | - | | - | | T | | - | | | | | C | - | | - | | - | | - | | - | |  | |
| MBohicon27a | | | C | - | | A | |  | | - | | - | | A | | - | | - | | T | | A | | C | | C | | | - | | - | | - | | - | | |  | | - | | - | | - | | - | | - | | - | | - | | G | | - | | - | | - | C | | - | | - | | - | | - | | - | | | | | C | - | | - | | - | | - | | - | |  | |
| MBohicon27b | | | - | - | | A | |  | | - | | - | | A | | C | | - | | * | | * | | * | | C | | | A | | - | | - | | - | | |  | | - | | - | | - | | - | | - | | - | | - | | G | | - | | - | | - | C | | - | | - | | - | | - | | - | | | | | C | - | | - | | - | | - | | - | |  | |
| MBohicon72a | | | C | - | | A | |  | | C | | - | | A | | - | | - | | * | | * | | * | | C | | | - | | - | | - | | - | | |  | | - | | - | | - | | - | | - | | - | | - | | G | | - | | A | | - | C | | - | | - | | - | | - | | - | | | | | - | - | | - | | - | | - | | - | |  | |
| MBohicon72b | | | - | - | | - | |  | | - | | G | | A | | - | | - | | * | | * | | * | | - | | | - | | - | | - | | - | | |  | | - | | - | | - | | - | | - | | - | | - | | - | | - | | - | | - | C | | - | | - | | - | | - | | - | | | | | - | - | | - | | - | | - | | - | |  | |
| MBoromo10a | | | - | - | | A | |  | | - | | - | | A | | - | | - | | * | | * | | * | | C | | | - | | - | | - | | - | | |  | | - | | - | | - | | - | | - | | - | | - | | - | | - | | - | | - | C | | - | | - | | T | | - | | - | | | | | - | - | | - | | - | | - | | - | |  | |
| MBoromo10b | | | C | - | | A | |  | | - | | - | | A | | - | | - | | T | | A | | C | | C | | | - | | - | | - | | - | | |  | | - | | - | | - | | - | | - | | - | | - | | - | | - | | - | | - | C | | - | | A | | - | | - | | - | | | | | - | - | | - | | - | | - | | - | |  | |
| MBoromo9a | | | C | - | | A | |  | | C | | - | | A | | - | | - | | * | | * | | * | | C | | | - | | - | | - | | - | | |  | | - | | - | | - | | - | | - | | - | | - | | - | | - | | - | | - | C | | - | | - | | - | | - | | - | | | | | - | - | | - | | - | | - | | - | |  | |
| MBoromo9b | | | - | - | | A | |  | | - | | - | | A | | C | | - | | * | | * | | * | | C | | | - | | - | | - | | - | | |  | | - | | - | | - | | - | | T | | - | | - | | - | | T | | C | | - | C | | A | | - | | - | | - | | - | | | | | - | - | | - | | - | | - | | - | |  | |
| MNiamoue18 | | | C | - | | A | |  | | - | | - | | A | | - | | - | | * | | * | | * | | - | | | - | | - | | - | | - | | |  | | - | | - | | T | | - | | - | | - | | - | | - | | - | | - | | A | C | | - | | - | | - | | - | | - | | | | | C | - | | - | | - | | - | | - | |  | |
| MNiamoue19 | | | C | - | | A | |  | | C | | - | | A | | - | | - | | * | | * | | * | | C | | | - | | - | | - | | - | | |  | | - | | - | | - | | - | | - | | - | | - | | - | | - | | - | | - | C | | A | | - | | - | | - | | - | | | | | C | - | | - | | - | | - | | - | |  | |
| MSeguere37a | | | - | A | | A | |  | | A | | - | | A | | C | | - | | * | | * | | * | | C | | | - | | - | | - | | - | | |  | | - | | - | | - | | - | | - | | - | | - | | - | | T | | C | | - | C | | A | | - | | - | | - | | - | | | | | - | - | | - | | - | | - | | - | |  | |
| MSeguere37b | | | C | - | | A | |  | | - | | - | | A | | - | | G | | T | | A | | C | | C | | | - | | - | | - | | T | | |  | | - | | - | | - | | - | | - | | - | | - | | - | | - | | - | | - | C | | - | | - | | - | | - | | - | | | | | C | - | | - | | - | | - | | - | |  | |
| MSeguere35a | | | C | - | | A | |  | | - | | - | | A | | - | | - | | * | | * | | * | | C | | | - | | - | | - | | - | | |  | | - | | - | | - | | - | | - | | - | | - | | - | | - | | - | | - | C | | - | | - | | - | | - | | - | | | | | C | - | | - | | - | | - | | - | |  | |
| MSeguere35b | | | - | - | | A | |  | | - | | - | | A | | C | | - | | * | | * | | * | | C | | | A | | - | | - | | - | | |  | | - | | - | | - | | T | | - | | - | | - | | - | | - | | - | | - | C | | - | | - | | - | | T | | - | | | | | C | - | | - | | - | | - | | - | |  | |
| MToumbokro2a | | | A | - | | A | |  | | - | | - | | A | | C | | - | | * | | * | | * | | C | | | - | | - | | - | | - | | |  | | - | | - | | - | | - | | - | | - | | - | | - | | - | | - | | - | C | | - | | - | | - | | - | | - | | | | | - | - | | - | | - | | - | | - | |  | |
| MToumbokro2b | | | - | - | | - | |  | | - | | - | | A | | - | | - | | * | | * | | * | | - | | | - | | - | | - | | - | | |  | | - | | - | | - | | - | | - | | **A** | | - | | - | | - | | - | | - | C | | - | | - | | - | | - | | - | | | | | - | - | | - | | - | | - | | - | |  | |
| MZogbodomey14a | | | C | - | | A | |  | | - | | - | | A | | - | | - | | * | | * | | * | | C | | | - | | A | | - | | - | | |  | | - | | - | | - | | - | | - | | - | | - | | - | | - | | - | | - | C | | - | | - | | - | | T | | - | | | | | C | - | | - | | - | | - | | - | |  | |
| MZogbodomey14b | | | C | - | | A | |  | | - | | - | | A | | C | | - | | * | | * | | * | | C | | | - | | - | | - | | - | | |  | | - | | - | | - | | - | | - | | - | | - | | - | | - | | - | | - | C | | - | | - | | - | | - | | - | | | | | - | - | | - | | - | | - | | - | |  | |
| MZogbodomey18a | | | C | - | | A | |  | | C | | - | | A | | - | | - | | * | | * | | * | | C | | | - | | - | | - | | - | | |  | | - | | - | | - | | - | | - | | - | | - | | - | | - | | - | | - | C | | A | | - | | - | | - | | - | | | | | - | - | | - | | - | | - | | - | |  | |
| MZogbodomey18b | | | C | - | | A | |  | | - | | - | | A | | - | | - | | * | | * | | * | | C | | | - | | - | | - | | - | | |  | | - | | - | | - | | - | | - | | - | | - | | - | | - | | - | | - | C | | - | | - | | - | | - | | - | | | | | - | - | | - | | - | | - | | - | |  | |
| MZogbodomey1a | | | C | - | | A | |  | | - | | - | | A | | - | | - | | * | | * | | * | | C | | | - | | - | | - | | - | | |  | | - | | - | | - | | - | | - | | - | | - | | G | | - | | - | | - | C | | A | | - | | - | | - | | - | | | | | - | - | | - | | - | | - | | - | |  | |
| MZogbodomey1b | | | - | - | | A | |  | | - | | - | | A | | C | | - | | * | | * | | * | | C | | | - | | - | | - | | - | | |  | | - | | - | | - | | - | | - | | - | | - | | - | | - | | - | | - | C | | - | | - | | - | | - | | - | | | | | - | - | | - | | - | | - | | - | |  | |
| SAbomey19 | | | - | - | | - | |  | | - | | - | | A | | - | | - | | * | | * | | * | | - | | | - | | - | | A | | - | | |  | | - | | T | | - | | - | | - | | - | | - | | - | | - | | - | | - | C | | - | | - | | - | | - | | - | | | | | C | - | | - | | C | | - | | - | |  | |
| SAbomey34a | | | C | - | | A | |  | | - | | - | | A | | - | | - | | T | | A | | C | | C | | | - | | - | | - | | - | | |  | | - | | - | | - | | - | | - | | - | | - | | G | | - | | - | | - | C | | - | | - | | - | | - | | - | | | | | - | - | | - | | - | | - | | - | |  | |
| SAbomey34b | | | C | - | | A | |  | | - | | - | | A | | - | | - | | * | | * | | * | | C | | | - | | - | | - | | - | | |  | | - | | - | | - | | - | | - | | - | | - | | - | | T | | C | | - | C | | A | | A | | - | | - | | - | | | | | - | G | | - | | - | | - | | - | |  | |
| SAbomey53 | | | C | - | | A | |  | | - | | - | | A | | - | | - | | * | | * | | * | | C | | | - | | - | | - | | - | | |  | | - | | - | | - | | - | | - | | - | | - | | - | | - | | - | | - | C | | - | | - | | - | | - | | - | | | | | - | - | | - | | - | | - | | - | |  | |
| SAbomey60 | | | C | - | | A | |  | | - | | - | | A | | - | | - | | T | | A | | C | | C | | | - | | - | | - | | - | | |  | | - | | - | | - | | - | | - | | - | | - | | - | | - | | - | | - | C | | - | | - | | - | | - | | - | | | | | - | - | | - | | - | | - | | - | |  | |
| SAbomey71 | | | C | - | | A | |  | | - | | - | | A | | - | | - | | * | | * | | * | | C | | | - | | - | | - | | - | | |  | | - | | - | | - | | - | | - | | - | | T | | - | | - | | C | | - | C | | - | | - | | - | | - | | - | | | | | - | - | | - | | - | | - | | - | |  | |
| SBohicon16 | | | C | - | | A | |  | | - | | - | | A | | - | | - | | T | | A | | C | | C | | | - | | - | | - | | - | | |  | | - | | - | | - | | - | | - | | - | | - | | - | | - | | - | | - | C | | - | | - | | - | | - | | - | | | | | C | G | | - | | - | | - | | - | |  | |
| SBohicon49 | | | C | - | | A | |  | | - | | - | | A | | - | | - | | * | | * | | * | | - | | | - | | - | | - | | - | | |  | | - | | - | | T | | - | | - | | - | | - | | - | | - | | T | | - | C | | - | | - | | - | | - | | - | | | | | - | - | | - | | - | | - | | - | |  | |
| SBoromo26 | | | C | - | | A | |  | | - | | - | | A | | - | | - | | * | | * | | * | | C | | | - | | - | | - | | - | | |  | | - | | - | | - | | - | | - | | - | | T | | - | | - | | C | | - | C | | - | | - | | - | | - | | - | | | | | - | - | | - | | - | | - | | - | |  | |
| SPaouignan37 | | | - | - | | - | |  | | - | | - | | A | | - | | - | | * | | * | | * | | - | | | - | | - | | - | | - | | |  | | - | | - | | - | | - | | - | | - | | - | | - | | - | | - | | - | C | | - | | - | | - | | - | | - | | | | | - | - | | - | | - | | - | | - | |  | |
| SPaouignan50 | | | C | - | | A | |  | | - | | - | | A | | - | | - | | * | | * | | * | | C | | | - | | - | | - | | - | | |  | | - | | - | | - | | - | | - | | - | | - | | - | | - | | - | | - | C | | - | | - | | - | | - | | A | | | | | C | - | | - | | - | | - | | - | |  | |
| SPaouignan52a | | | - | - | | A | |  | | - | | - | | - | | - | | - | | * | | * | | * | | C | | | - | | - | | - | | - | | |  | | - | | - | | - | | - | | - | | - | | - | | G | | - | | - | | - | C | | - | | - | | - | | - | | - | | | | | C | - | | - | | - | | - | | - | |  | |
| SPaouignan52b | | | C | - | | A | |  | | - | | - | | A | | - | | - | | T | | A | | C | | C | | | - | | - | | - | | - | | |  | | - | | - | | - | | - | | - | | - | | - | | - | | - | | C | | - | C | | - | | - | | - | | - | | - | | | | | C | - | | - | | - | | - | | - | |  | |
| Sseguere4 | | | - | - | | A | |  | | - | | - | | A | | - | | - | | * | | * | | * | | C | | | - | | - | | - | | - | | |  | | - | | - | | - | | - | | - | | - | | - | | - | | - | | - | | - | C | | - | | - | | - | | - | | - | | | | | - | - | | - | | - | | - | | - | |  | |
| SSeguere1 | | | C | - | | A | |  | | - | | - | | A | | - | | - | | T | | A | | C | | C | | | - | | - | | - | | - | | |  | | - | | - | | - | | - | | - | | - | | - | | - | | - | | - | | - | C | | - | | - | | - | | - | | - | | | | | - | - | | - | | - | | - | | - | |  | |
| SSeguere2a | | | - | - | | - | |  | | - | | - | | - | | - | | - | | * | | * | | * | | - | | | - | | - | | - | | - | | |  | | - | | T | | - | | - | | - | | - | | - | | - | | - | | - | | - | C | | - | | - | | - | | - | | - | | | | | C | G | | - | | - | | - | | - | |  | |
| SSeguere2b | | | - | - | | - | |  | | - | | - | | A | | - | | - | | * | | * | | * | | - | | | - | | - | | - | | - | | |  | | - | | - | | T | | - | | - | | - | | - | | - | | - | | - | | - | C | | - | | - | | - | | - | | - | | | | | - | G | | - | | - | | - | | - | |  | |
| SSeguere48a | | | - | - | | - | |  | | - | | - | | A | | - | | - | | T | | A | | C | | C | | | - | | - | | - | | - | | |  | | - | | - | | - | | - | | - | | - | | - | | - | | - | | C | | - | C | | - | | - | | - | | - | | - | | | | | C | - | | - | | - | | - | | - | |  | |
| SDoulassoba14 | | | C | - | | A | |  | | - | | - | | A | | - | | - | | * | | * | | * | | C | | | - | | - | | - | | - | | |  | | - | | - | | - | | - | | - | | - | | - | | - | | T | | C | | - | C | | A | | - | | - | | - | | - | | | | | - | G | | - | | - | | - | | - | |  | |
| SDoulassoba35a | | | - | - | | A | |  | | - | | - | | A | | - | | - | | * | | * | | * | | C | | | - | | - | | - | | - | | |  | | - | | - | | - | | - | | - | | - | | - | | - | | - | | - | | - | C | | - | | - | | - | | - | | - | | | | | - | G | | - | | - | | - | | - | |  | |
| SZogbodomey17 | | | - | - | | - | |  | | - | | - | | A | | - | | - | | * | | * | | * | | - | | | - | | - | | A | | - | | |  | | - | | T | | - | | - | | - | | - | | - | | - | | - | | - | | - | C | | - | | - | | - | | - | | - | | | | | - | - | | - | | - | | - | | - | |  | |
|  | Exon 3 | | | | | | | | | | | | | | | | | | | | | | | | |  | | Intron 3 | | | | | | | | | | | | | | | | | | | | | | | | | | | | | | | | | | | | | | | | | | | | |  | | Exon 4 | | | | | | | | | | | | | |
|  | | 422 | | | 427 | | 444 | | 452 | | 464 | | 470 | | 491 | | 497 | | 503 | | 518 | | 534 | |  | | 9 | | | 10 | | 11 | | 15 | | | 18 | | | | 22 | | 23 | | 25 | | 26 | | 27 | | 28 | | 29 | | 31 | | 48 | | 49 | | | 52 | | 57 | | 65 | | 70 | | 77 | |  | | 1 | | | | 19 | | 22 | | 34 | | 36 | | 46 | | 57 |
| Kisumu | | G | | | A | | C | | C | | T | | C | | G | | G | | C | | C | | C | |  | | C | | | T | | T | | - | | | G | | | | A | | A | | G | | A | | G | | G | | G | | A | | T | | G | | | C | | T | | C | | T | | C | |  | | C | | | | T | | C | | T | | A | | T | | T |
| S&M-R | | - | | | - | | - | | - | | - | | - | | C | | - | | - | | - | | - | |  | | - | | | - | | - | | G | | | T | | | | T | | C | | - | | T | | A | | - | | T | | - | | - | | - | | | - | | - | | - | | - | | - | |  | | - | | | | - | | - | | - | | - | | C | | - |
| Arabiensis1 | | - | | | - | | - | | - | | - | | - | | - | | - | | - | | - | | - | |  | | - | | | - | | - | | - | | | T | | | | - | | - | | - | | - | | - | | - | | C | | T | | - | | - | | | - | | - | | - | | - | | - | |  | | - | | | | - | | - | | - | | - | | - | | - |
| Arabiensis2 | | - | | | - | | - | | - | | - | | - | | - | | - | | - | | - | | - | |  | | T | | | - | | - | | - | | | T | | | | - | | G | | - | | - | | - | | - | | C | | T | | - | | - | | | A | | - | | - | | - | | - | |  | | - | | | | - | | - | | C | | - | | - | | - |
| MBoromo41 | | - | | | - | | - | | - | | - | | - | | - | | - | | - | | - | | - | |  | | - | | | - | | - | | - | | | T | | | | - | | - | | - | | - | | - | | A | | - | | - | | - | | A | | | - | | - | | - | | - | | - | |  | | - | | | | - | | - | | C | | - | | - | | - |
| MBohicon27a | | - | | | - | | - | | - | | - | | T | | - | | A | | - | | - | | - | |  | | - | | | - | | - | | - | | | T | | | | - | | - | | - | | - | | - | | A | | - | | - | | - | | - | | | - | | - | | - | | - | | T | |  | | - | | | | - | | - | | - | | - | | - | | - |
| MBohicon27b | | - | | | - | | - | | - | | - | | - | | - | | - | | - | | - | | - | |  | | - | | | - | | - | | - | | | - | | | | - | | - | | - | | - | | - | | - | | - | | - | | - | | - | | | - | | - | | - | | - | | - | |  | | - | | | | C | | - | | - | | - | | - | | - |
| MBohicon72a | | - | | | **G** | | - | | - | | - | | - | | - | | - | | - | | - | | - | |  | | - | | | - | | - | | - | | | T | | | | - | | - | | - | | - | | - | | - | | - | | - | | - | | - | | | - | | - | | - | | - | | - | |  | | - | | | | - | | - | | C | | - | | - | | - |
| MBohicon72b | | - | | | - | | - | | - | | - | | - | | - | | - | | - | | - | | - | |  | | - | | | - | | - | | - | | | - | | | | - | | - | | - | | - | | - | | - | | - | | - | | - | | - | | | - | | - | | - | | - | | - | |  | | - | | | | - | | - | | C | | - | | - | | - |
| MBoromo10a | | - | | | - | | - | | - | | - | | - | | - | | - | | - | | - | | - | |  | | - | | | - | | - | | - | | | - | | | | - | | - | | - | | - | | - | | - | | - | | - | | - | | - | | | - | | - | | - | | - | | - | |  | | - | | | | - | | - | | C | | - | | - | | - |
| MBoromo10b | | - | | | - | | - | | - | | - | | - | | - | | - | | - | | - | | - | |  | | - | | | - | | - | | - | | | T | | | | - | | - | | - | | - | | - | | - | | - | | - | | - | | - | | | - | | - | | - | | - | | - | |  | | - | | | | - | | - | | C | | - | | - | | - |
| MBoromo9a | | A | | | - | | - | | - | | - | | - | | - | | - | | - | | - | | - | |  | | - | | | - | | - | | - | | | T | | | | - | | - | | - | | - | | - | | - | | - | | - | | - | | - | | | - | | - | | - | | - | | - | |  | | - | | | | - | | - | | C | | - | | - | | - |
| MBoromo9b | | - | | | - | | - | | - | | - | | - | | - | | - | | - | | - | | - | |  | | - | | | - | | - | | - | | | T | | | | - | | - | | - | | - | | - | | - | | - | | - | | - | | - | | | - | | - | | - | | - | | - | |  | | - | | | | - | | - | | C | | - | | A | | - |
| MNiamoue18 | | - | | | - | | - | | - | | - | | - | | - | | - | | - | | - | | - | |  | | - | | | - | | C | | - | | | - | | | | - | | - | | - | | - | | - | | - | | - | | - | | - | | - | | | - | | - | | - | | - | | - | |  | | - | | | | - | | - | | - | | - | | - | | - |
| MNiamoue19 | | - | | | - | | - | | - | | - | | - | | - | | - | | - | | - | | - | |  | | - | | | - | | - | | - | | | T | | | | - | | G | | - | | - | | - | | - | | C | | T | | - | | - | | | - | | - | | - | | - | | - | |  | | - | | | | - | | - | | - | | **T** | | - | | - |
| MSeguere37a | | - | | | - | | - | | - | | - | | - | | - | | - | | - | | - | | T | |  | | - | | | - | | C | | - | | | - | | | | - | | - | | - | | - | | - | | - | | - | | - | | - | | - | | | - | | - | | - | | - | | - | |  | | - | | | | - | | - | | C | | - | | - | | - |
| MSeguere37b | | - | | | - | | - | | T | | - | | - | | - | | - | | T | | - | | - | |  | | - | | | - | | - | | G | | | T | | | | T | | C | | - | | T | | A | | - | | T | | - | | - | | - | | | - | | - | | - | | - | | - | |  | | - | | | | - | | T | | - | | - | | - | | - |
| MSeguere35a | | - | | | - | | - | | - | | - | | - | | - | | - | | - | | - | | - | |  | | - | | | - | | - | | - | | | - | | | | - | | - | | - | | - | | - | | - | | - | | - | | - | | - | | | - | | - | | - | | - | | - | |  | | - | | | | C | | - | | - | | - | | - | | - |
| MSeguere35b | | - | | | - | | - | | - | | - | | - | | - | | - | | - | | T | | - | |  | | - | | | - | | - | | - | | | T | | | | - | | - | | - | | - | | - | | A | | - | | - | | - | | - | | | - | | - | | - | | - | | - | |  | | - | | | | - | | - | | C | | - | | - | | - |
| MToumbokro2Sa | | - | | | - | | - | | - | | - | | - | | - | | - | | - | | - | | - | |  | | - | | | - | | - | | - | | | - | | | | - | | - | | - | | - | | - | | - | | - | | - | | - | | - | | | - | | - | | - | | - | | - | |  | | - | | | | - | | - | | C | | - | | - | | - |
| MToumbokro2Sb | | - | | | - | | - | | - | | - | | - | | - | | - | | - | | - | | - | |  | | - | | | - | | - | | - | | | - | | | | - | | - | | - | | - | | - | | - | | - | | - | | - | | - | | | - | | - | | - | | - | | - | |  | | - | | | | - | | - | | - | | - | | - | | - |
| MZogbodomey14a | | - | | | - | | - | | - | | - | | - | | - | | - | | - | | - | | - | |  | | - | | | - | | - | | - | | | T | | | | - | | - | | - | | - | | - | | - | | - | | - | | - | | - | | | - | | - | | - | | - | | - | |  | | - | | | | - | | - | | C | | - | | - | | - |
| MZogbodomey14b | | - | | | - | | - | | - | | - | | - | | - | | - | | - | | - | | - | |  | | - | | | - | | - | | - | | | T | | | | - | | - | | - | | - | | - | | - | | - | | - | | - | | - | | | - | | - | | - | | - | | - | |  | | - | | | | - | | - | | C | | - | | - | | - |
| MZogbodomey18a | | - | | | - | | - | | - | | - | | - | | - | | - | | - | | - | | - | |  | | - | | | - | | - | | - | | | T | | | | - | | - | | - | | - | | - | | - | | - | | - | | - | | - | | | - | | - | | - | | - | | - | |  | | - | | | | - | | - | | C | | - | | A | | - |
| MZogbodomey18b | | - | | | - | | - | | - | | - | | - | | - | | - | | - | | - | | - | |  | | - | | | A | | - | | - | | | T | | | | - | | G | | - | | - | | - | | - | | C | | T | | - | | - | | | - | | - | | - | | - | | - | |  | | - | | | | - | | - | | C | | - | | - | | - |
| MZogbodomey1a | | - | | | - | | - | | - | | - | | - | | - | | - | | - | | - | | - | |  | | - | | | - | | - | | - | | | T | | | | - | | - | | - | | - | | - | | A | | - | | - | | - | | - | | | - | | - | | - | | - | | - | |  | | - | | | | - | | - | | C | | - | | - | | - |
| MZogbodomey1b | | - | | | - | | - | | T | | - | | - | | - | | - | | T | | - | | - | |  | | - | | | - | | - | | G | | | T | | | | T | | C | | - | | T | | A | | - | | T | | - | | - | | - | | | - | | - | | - | | - | | - | |  | | - | | | | - | | T | | - | | - | | - | | - |
| SAbomey19 | | - | | | - | | T | | - | | - | | - | | - | | - | | - | | - | | - | |  | | T | | | - | | - | | - | | | T | | | | - | | G | | - | | - | | - | | - | | C | | T | | - | | - | | | - | | - | | - | | - | | - | |  | | - | | | | - | | - | | C | | - | | - | | - |
| SAbomey34a | | - | | | - | | - | | - | | - | | - | | - | | - | | - | | - | | - | |  | | - | | | - | | - | | - | | | T | | | | - | | - | | - | | - | | - | | - | | - | | - | | - | | - | | | - | | - | | - | | - | | - | |  | | - | | | | - | | - | | C | | - | | - | | - |
| SAbomey34b | | - | | | - | | - | | - | | C | | - | | - | | - | | - | | - | | - | |  | | - | | | - | | - | | - | | | T | | | | - | | - | | - | | - | | - | | - | | - | | - | | - | | - | | | - | | - | | - | | - | | - | |  | | - | | | | - | | T | | - | | - | | - | | - |
| SAbomey53 | | - | | | - | | - | | - | | - | | - | | - | | - | | - | | - | | - | |  | | - | | | - | | - | | - | | | T | | | | - | | - | | - | | - | | - | | A | | - | | - | | - | | - | | | - | | - | | - | | - | | - | |  | | - | | | | - | | - | | C | | - | | - | | - |
| SAbomey60 | | - | | | - | | - | | - | | - | | - | | - | | - | | - | | - | | - | |  | | - | | | - | | - | | - | | | T | | | | - | | - | | - | | - | | - | | - | | - | | - | | - | | - | | | - | | - | | - | | - | | - | |  | | - | | | | - | | - | | - | | - | | - | | - |
| SAbomey71 | | - | | | - | | - | | - | | - | | - | | - | | - | | - | | - | | - | |  | | - | | | - | | - | | - | | | T | | | | - | | G | | - | | G | | - | | - | | C | | T | | - | | - | | | - | | - | | - | | C- | | - | |  | | - | | | | - | | - | | - | | - | | - | | - |
| SBohicon16 | | - | | | - | | - | | - | | - | | - | | - | | - | | - | | - | | - | |  | | - | | | - | | - | | - | | | - | | | | - | | - | | - | | - | | - | | - | | - | | - | | - | | - | | | - | | - | | - | | - | | - | |  | | A | | | | C | | - | | - | | - | | - | | - |
| SBohicon49 | | - | | | - | | - | | - | | - | | - | | - | | - | | - | | - | | - | |  | | - | | | - | | - | | - | | | T | | | | - | | G | | T | | - | | - | | - | | C | | T | | - | | - | | | - | | - | | - | | C- | | - | |  | | - | | | | - | | - | | - | | - | | - | | - |
| SBoromo26 | | - | | | - | | - | | - | | - | | - | | - | | - | | - | | - | | - | |  | | - | | | - | | - | | - | | | T | | | | - | | G | | - | | - | | - | | - | | C | | T | | - | | - | | | - | | - | | - | | C- | | - | |  | | - | | | | - | | - | | - | | - | | - | | - |
| SPaouignan37 | | - | | | - | | - | | - | | - | | - | | - | | - | | - | | - | | - | |  | | - | | | - | | - | | - | | | T | | | | - | | - | | - | | - | | - | | - | | - | | - | | - | | - | | | - | | C | | - | | - | | - | |  | | - | | | | - | | - | | C | | - | | - | | G |
| SPaouignan50 | | - | | | - | | - | | - | | - | | - | | - | | - | | - | | - | | - | |  | | - | | | - | | C | | - | | | - | | | | - | | - | | - | | - | | - | | - | | - | | - | | - | | - | | | - | | - | | - | | - | | - | |  | | - | | | | - | | - | | C | | - | | - | | - |
| SPaouignan52a | | - | | | - | | - | | - | | - | | - | | - | | - | | - | | - | | - | |  | | - | | | - | | - | | - | | | T | | | | - | | - | | - | | - | | - | | - | | - | | - | | - | | - | | | - | | - | | - | | - | | - | |  | | - | | | | - | | - | | C | | - | | - | | - |
| SPaouignan52b | | - | | | - | | - | | - | | - | | - | | - | | - | | - | | - | | - | |  | | - | | | A | | - | | - | | | T | | | | - | | G | | - | | - | | - | | - | | C | | T | | - | | - | | | - | | - | | - | | - | | - | |  | | - | | | | - | | - | | C | | - | | - | | - |
| SSeguere4 | | - | | | - | | - | | - | | - | | - | | - | | - | | - | | - | | - | |  | | - | | | - | | - | | - | | | T | | | | - | | - | | - | | - | | - | | A | | - | | - | | - | | - | | | - | | - | | A | | - | | - | |  | | - | | | | - | | - | | C | | - | | - | | - |
| SSeguere1 | | - | | | - | | - | | - | | C | | T | | - | | - | | - | | - | | - | |  | | - | | | - | | - | | - | | | T | | | | - | | - | | - | | - | | - | | - | | - | | - | | - | | - | | | - | | - | | - | | - | | - | |  | | - | | | | - | | - | | C | | - | | - | | - |
| SSeguere2a | | - | | | - | | - | | - | | - | | - | | - | | - | | - | | - | | - | |  | | - | | | - | | - | | - | | | T | | | | - | | - | | - | | - | | - | | - | | C | | T | | - | | - | | | - | | - | | - | | - | | - | |  | | - | | | | - | | - | | C | | - | | - | | - |
| SSeguere2b | | - | | | - | | - | | - | | - | | - | | - | | - | | - | | - | | - | |  | | - | | | - | | - | | - | | | T | | | | - | | G | | - | | - | | - | | - | | C | | T | | C | | - | | | - | | - | | - | | - | | - | |  | | - | | | | - | | - | | - | | - | | - | | - |
| SSeguere48a | | - | | | - | | - | | - | | - | | - | | - | | - | | - | | - | | - | |  | | - | | | - | | - | | - | | | T | | | | - | | - | | - | | - | | - | | A | | - | | - | | - | | - | | | - | | - | | A | | - | | - | |  | | - | | | | - | | - | | C | | - | | - | | - |
| SDoulassoba14 | | - | | | - | | - | | - | | - | | - | | - | | - | | - | | - | | - | |  | | - | | | - | | C | | - | | | - | | | | - | | - | | - | | - | | - | | - | | - | | - | | - | | - | | | - | | - | | - | | - | | - | |  | | - | | | | - | | - | | C | | - | | - | | - |
| SDoulassoba35 | | - | | | - | | - | | - | | - | | - | | - | | - | | - | | - | | - | |  | | - | | | - | | - | | - | | | T | | | | - | | - | | - | | - | | - | | A | | - | | - | | - | | A | | | - | | - | | - | | - | | - | |  | | - | | | | - | | - | | C | | - | | - | | - |
| SZogbodomey17 | | - | | | - | | - | | - | | - | | - | | - | | - | | - | | - | | - | |  | | - | | | - | | - | | - | | | T | | | | - | | - | | - | | - | | - | | - | | - | | - | | - | | - | | | - | | - | | - | | - | | - | |  | | - | | | | - | | - | | - | | - | | C | | - |

**Table S1**: Susceptible and resistant allele sequences

Comparisons of sequences of all susceptible alleles and the single resistant allele sequence (S&M-R). Identical alleles were removed from Table. The first sequence (Kisumu) is the reference. The dashes (-) and the stars (*) indicate identity or a deletion, respectively. The position from the first nucleotide of each part of the sequenced fragment is indicated at the top. The G119S mutation at position 363 of exon 3, distinguishing R and S copies is indicated in bold. The three other mutations leading to an amino acid substitution are highlighted in grey.
